# NeuroSmog: Determining the Impact of Air Pollution on the Developing Brain: Project Protocol

**DOI:** 10.3390/ijerph19010310

**Published:** 2021-12-28

**Authors:** Iana Markevych, Natasza Orlov, James Grellier, Katarzyna Kaczmarek-Majer, Małgorzata Lipowska, Katarzyna Sitnik-Warchulska, Yarema Mysak, Clemens Baumbach, Maja Wierzba-Łukaszyk, Munawar Hussain Soomro, Mikołaj Compa, Bernadetta Izydorczyk, Krzysztof Skotak, Anna Degórska, Jakub Bratkowski, Bartosz Kossowski, Aleksandra Domagalik, Marcin Szwed

**Affiliations:** 1 Institute of Psychology, Jagiellonian University, Ingardena 6, 30-060 Krakow, Poland; iana.markevych@uj.edu.pl (I.M.); natasza.orlov@uj.edu.pl (N.O.); J.Grellier@exeter.ac.uk (J.G.); yaremamysak@gmail.com (Y.M.); clemens.baumbach@uj.edu.pl (C.B.); majamwierzba@gmail.com (M.W.-Ł.); munawar_soomro@hotmail.com (M.H.S.); mikolaj.compa@doctoral.uj.edu.pl (M.C.); bernadetta.izydorczyk@uj.edu.pl (B.I.); 2 Department of Psychosis Studies, Institute of Psychiatry, Psychology and Neuroscience, King’s College London, De Crespigny Park, London SE5 8AF, UK; 3 European Centre of Environment and Human Health, University of Exeter Medical School, Royal Cornwall Hospital, Truro, Cornwall TR1 3HD, UK; 4 Institute of Environmental Protection-National Research Institute, Krucza 5/11d, 00-548 Warsaw, Poland; K.Kaczmarek@ibspan.waw.pl (K.K.-M.); krzysztof.skotak@ios.edu.pl (K.S.); anna.degorska@ios.edu.pl (A.D.); jakub.bratkowski@ios.edu.pl (J.B.); 5 Systems Research Institute, Polish Academy of Sciences, Newelska 6, 01-447 Warsaw, Poland; 6 Faculty of Management and Social Communication, Institute of Applied Psychology, Jagiellonian University, Łojasiewicza 4, 30-348 Krakow, Poland; malgorzata.lipowska@ug.edu.pl (M.L.); katarzyna.sitnik-warchulska@uj.edu.pl (K.S.-W.); 7 Institute of Psychology, University of Gdansk, Bażyńskiego 4, 80-952 Gdansk, Poland; 8 ENIANO GmbH, Schwanthalerstraße 73, 80336 Munich, Germany; 9 Laboratory of Brain Imaging, Nencki Institute for Experimental Biology, Pasteur 3, 02-093 Warsaw, Poland; b.kossowski@nencki.edu.pl; 10 Brain Imaging Core Facility, Małopolska Centre of Biotechnology, Jagiellonian University, Gronostajowa 7A, 30-387 Krakow, Poland; aleksandra.domagalik@uj.edu.pl

**Keywords:** PM_2.5_, PM_10_, children, neuroimaging, cognitive functioning, social functioning, epidemiology, Poland, air pollution, case–control study

## Abstract

Exposure to airborne particulate matter (PM) may affect neurodevelopmental outcomes in children. The mechanisms underlying these relationships are not currently known. We aim to assess whether PM affects the developing brains of schoolchildren in Poland, a country characterized by high levels of PM pollution. Children aged from 10 to 13 years (*n* = 800) are recruited to participate in this case–control study. Cases (children with attention deficit hyperactivity disorder (ADHD)) are being recruited by field psychologists. Population-based controls are being sampled from schools. The study area comprises 18 towns in southern Poland characterized by wide-ranging levels of PM. Comprehensive psychological assessments are conducted to assess cognitive and social functioning. Participants undergo structural, diffusion-weighted, task, and resting-state magnetic resonance imaging (MRI). PM concentrations are estimated using land use regression models, incorporating information from air monitoring networks, dispersion models, and characteristics of roads and other land cover types. The estimated concentrations will be assigned to the prenatal and postnatal residential and preschool/school addresses of the study participants. We will assess whether long-term exposure to PM affects brain function, structure, and connectivity in healthy children and in those diagnosed with ADHD. This study will provide novel, in-depth understanding of the neurodevelopmental effects of PM pollution.

## 1. Introduction

Air pollution has received increased global public attention in recent years as presenting a major risk to human health [1]. According to the estimates of the World Health Organization (WHO), ambient air pollution accounts for 4.3 million deaths per year [2]. Exposure to outdoor particulate matter (PM) with an aerodynamic diameter <2.5 µm (PM_2.5_) is the fifth-ranking risk factor for mortality worldwide [3]. Epidemiological, pathophysiological, and animal model studies provide extensive evidence that short- and long-term exposure to particulate air pollution causes cardiovascular diseases [4,5], contributes to respiratory health deterioration and allergy development [6,7], and can lead to lung cancer [8,9]. Organic compounds and trace metals in PM may harm human health throughout the course of life, beginning in utero [10,11,12,13].

PM has been found incorporated within human brain tissue. Most likely, it enters via the olfactory and the gastrointestinal nerves, and is associated with abnormal protein aggregation in the brainstem [14,15]. In studies investigating the effects of PM using rodent models, inflammation, histopathological changes in the brain [16], and behavioural alterations [17,18] have been attributed to PM exposure. Two recent studies on mice and rats showed that astrocyte function and mitochondrial activity in the cortex were severely affected by PM; larger effects were observed for exposure to smaller particle sizes [19,20]. Some studies have shown that PM can affect the structure and the functioning of the human brain [21], but the results of this research are far from conclusive. For example, prenatal exposure to PM was negatively linked with cortical thickness in children aged between 6 and 10 years and was associated with impairment in inhibitory control [22]. Prenatal exposure to PM is also associated with decreased size of the corpus callosum and higher prevalence of behavioural problems in children aged between 8 and 12 years [23]. Postnatal air pollution exposure has been found to be associated with altered functional connectivity during rest [24]. Another study [17] has reported changes in grey matter surface area, volume, and thickness, but with no effect on cognitive functioning assessed by the comprehensive National Health Institute (NIH) Toolbox Cognitive Battery in children aged 9–10 years. Although exposure to PM has been reported to increase inattention, hyperactivity [12], and impulsivity symptoms [22]; lead to the lower intelligence quotient (IQ) [25]; and impact the prevalence of attention deficit hyperactivity disorder (ADHD) in children [26], the evidence for these effects is far from conclusive. In sum, the effects of PM and air pollution on neurodevelopment merit extensive further study.

Emissions of many air pollutants in the European Union (EU) have decreased over the past three decades, and air quality is gradually improving. In particular, between the years 2000 and 2014, significantly decreasing trends in annual average concentrations of PM with an aerodynamic diameter <10 µm (PM_10_) and nitrogen dioxide (NO_2_) were reported [27,28]. Despite these improvements, EU urban populations are exposed to PM levels that exceed the WHO limits for the protection of human health [29]. Between 2000 and 2010, the daily limit value for PM_2.5_ and PM_10_ concentrations in the EU were exceeded from 16 to 52% and from 18 to 44% of days, respectively [29]. These exceedances of PM limit values have mostly been observed in the eastern EU countries of Bulgaria, the Czech Republic, Slovakia, and Poland [28,29]. 

Most of the PM pollution in Poland results from the combustion of coal and other fossil fuels in power and heat generation, and from traffic emissions [30,31]. Estimates of the mortality attributable to PM_2.5_, based on air quality monitoring data for Poland, have demonstrated that the urban Polish population experiences an unduly high impact from particulate air pollution on their health [32]. Annual deaths attributable to air pollution in Poland were estimated at 39,800 for the year 2000, increasing to 47,300 deaths for the year 2017 [33]. 

The overall objective of the NeuroSmog study is to assess whether long-term exposure to outdoor PM affects brain function, structure, and connectivity in both healthy children and in those diagnosed with ADHD. Primarily, we will focus on the effect of PM on neural systems for attention and inhibitory control. In addition to neuroimaging, impacts of PM on these systems will be investigated using behavioural tests, including IQ. To meet the study aim, we assembled a multidisciplinary team of experts comprising air pollution modellers, environmental epidemiologists, neuroscientists, and clinical psychologists.

## 2. Methods

### 2.1. Study Area

**Figure 1:**
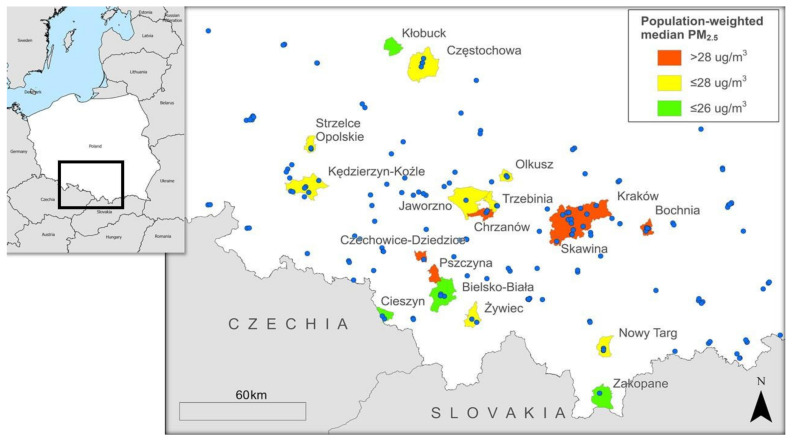
NeuroSmog study towns coloured according to their population-weighted median PM_2.5_ (2015) levels, as well as the locations of the air-monitoring stations used for air pollution modelling (blue dots), and location of Poland on the map of Europe (study area highlighted by a black square).

We selected towns in southern Poland, based on their levels of particulate air pollution and population size, that are located within two hours’ drive to the magnetic resonance imaging (MRI) scanning centre in Kraków. The particulate air pollution level of each candidate town (*n* = 202) was classified as high, medium, or low based on the population-weighted median air pollution level inferred from 1 km × 1 km interpolated PM_2.5_ data for the year 2015 [34]. We chose small and large towns across different levels of air pollution to minimize urbanicity-related confounding. Towns with more than 90,000 inhabitants were classified as large; others were classified as small. Accordingly, we selected 18 towns (Table 1, Figure 1). 

We are recruiting comparable numbers of children (cases and controls) for each combination of air pollution level and town population size (Table 1). Within each such combination, the number of children recruited per town is approximately proportional to the town’s population size. 

### 2.2. Study Design and Study Population

Recruitment of study participants began in October 2020, with the aim of having 800 children complete all study procedures by the end of 2022. We are recruiting one ADHD case (intended number *n* = 267) per two population controls (intended number *n* = 533).

Cases with ADHD diagnosis according to the 11th revision of the International Classification of Diseases and Related Health Problems (ICD-11) [35] are recruited by field psychologists in the study towns. Children at risk of ADHD are referred to the psychologists either by cooperative school psychologists or teachers, or by paediatricians, psychologists, psychiatrists, or neurologists from psychological-educational counselling centres and mental health centres. In addition, cases are also recruited from families who voluntarily contacted us after seeing our recruitment advertisements.

We use a two-step model to verify and eliminate ADHD diagnosis in cases and controls, respectively (see Section 2.6). Firstly, a comprehensive psychological assessment is administered and evaluated by field psychologists. Secondly, three consultant clinical psychologists review the assessments to verify the diagnoses according to the ICD-11. Cases that do not meet the ICD-11 criteria are excluded from the study; controls who meet the ADHD diagnostic criteria are recruited into the case arm. 

All study participants are fluent Polish speakers, aged between 10 and 13 years, have average or above average intelligence (i.e., attend non-specialised schools), are in grades IV–VI, and attend school in the selected towns. Exclusion criteria for all are diagnosed intellectual disability, neurological or comorbid psychiatric disorders, other serious medical conditions, as well as contraindications to MRI. Children born before the 35th week or after the 40th week of gestation, or with birth weight <2500 g or with an Apgar score <8 are also excluded. As we are interested in the long-term effects of air pollution, children who resided outside of Poland during the last year are also excluded, as are children whose legal guardians are not fluent in Polish. 

A team of field psychologists with at least five years of clinical experience was recruited to enlist potential cases and perform psychological evaluation of all children. Prior to beginning their work, all psychologists received accredited training in the use of psychological tests utilised in the study (see Section 2.6). They were also trained in the administration of two behavioural tests (see Section 2.7), as well as in data protection and privacy laws.

Population controls are recruited from randomly selected larger non-specialized primary schools within each of the study towns (one to five schools per town). Lists of candidate schools were retrieved from the Boards of Education (Kuratoria Oświaty). Within each of the selected schools, classes are randomly selected in grades IV–VI. Within each class, one to five pupils are selected at random. The sampling is defined a priori using randomized lists defining which classes in every grade and which pupils in every selected class to approach and in what order. The randomization was implemented using the *sample.int()* function in R statistical software [36]. 

Headteachers of selected schools are contacted and asked to support the study’s recruitment campaign and sign a formal collaborative agreement. Participating schools are promoted on the project website, and are offered workshops on neurodevelopment and on the study results. Consent for contact from the legal guardians is obtained by the schools. Families who give their consent are contacted by telephone by research assistants. The assistants explain the study aims and procedures, confirm study eligibility, and invite the legal guardians to participate in the study. 

If a school does not agree to participate, the next school in the randomized list of schools is contacted. If the legal guardians of a selected child do not consent to being contacted, the next family from the randomized list of pupils is contacted. Where insufficient numbers of pupils are recruited in each grade, additional pupils are selected from the next class in the randomized list of classes (for that grade) using the same procedure until the desired number of pupils are recruited.

### 2.3. Study Timeline Overview and Progress

All psychological and behavioural assessments are performed during three meetings with the field psychologist. Each meeting lasts about one hour and twenty minutes to two hours, and both the child and their legal guardian are present. At the final meeting, the results of the assessments are explained to the child and to their guardian, a formal psychological evaluation is provided. The MRI scan is completed in Kraków within three months of the psychological evaluation. Typical MRI sessions last up to one and a half hours. After the MRI session, families are rewarded with vouchers equal to 270 PLN (about 60 EUR at the time of writing) and are reimbursed for travel expenses. The children receive t-shirts printed with MRI images of their brains. 

**Figure 2:**
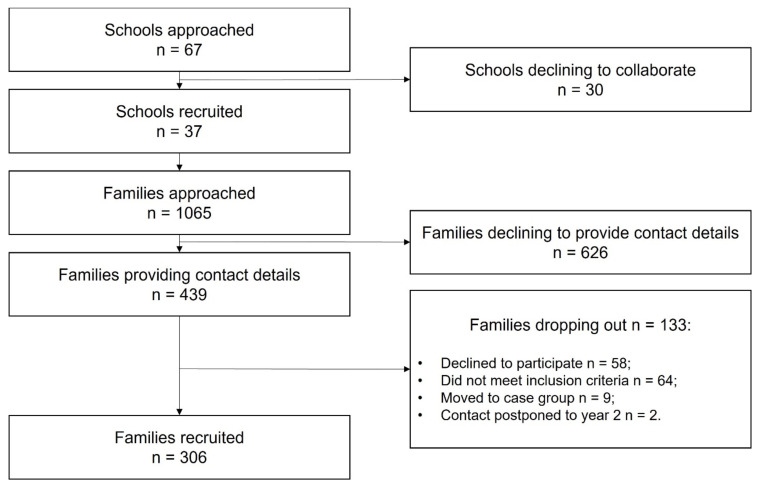
Flow chart describing the progress in the recruitment of controls at the end of the Year 1 (from October 2020 to June 2021).

Figure 2 illustrates the progress in the recruitment of controls at the end of the first study year (June 2021). 

**Figure 3:**
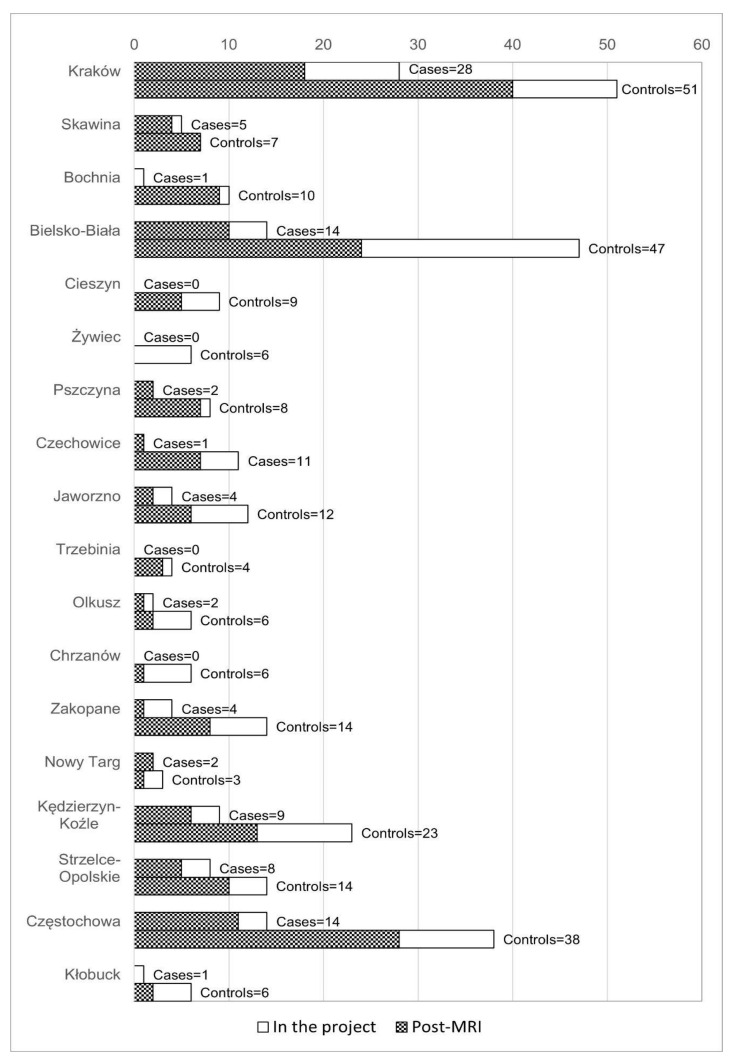
Town-level control-case-specific progress of testing children in the project as of 9 September 2021.

A total of 370 children have undergone psychological evaluation, of whom 236 have also completed the MRI scan. Figure 3 depicts the number of cases and controls tested in each town at the end of August 2021.

### 2.4. Data Management

Behavioural testing is conducted on HP laptops with 15.6” screens, set up identically for each field psychologist. The laptops are encrypted using BitLocker and synchronised with a secure OneDrive cloud folder at the Jagiellonian University that collects raw data from every test performed. 

To minimise the impact of the study on the environment and to reduce transcription from paper to computer, the psychological data are—wherever possible—collected digitally using online questionnaires implemented in survey tools developed by Qualtrics (https://www.qualtrics.com, accessed on 23 December 2021). 

The raw data are processed and prepared for import in R and Python. To allow easy data selection and extraction of variables of interest by data analysts, a secure user website will be implemented that will interface with an SQLite database.

### 2.5. Study-Specific Questionnaires

Two questionnaires have been developed for the study: a General Questionnaire, and an Address Questionnaire. The General Questionnaire is a Qualtrics-based tool filled in by a legal guardian that collects data on confounders, effect modifiers, and additional exposures. Some items were adopted from the existing studies [37,38,39,40], and some of the items were invented for the purpose of NeuroSmog. Briefly, information on socio-demographics, pregnancy and early life, general health, habits, academic performance, home, and neighbourhood environments of the child is being collected (see Supplementary File S1 for a paper-adopted version of the questionnaire). Non-response options are always presented as a variation of “not applicable”, “don’t know”, “don’t wish to respond”, and at the same time, all responses are forced. The General Questionnaire was validated on a small sample of volunteers. 

The Address Questionnaire collects full prenatal and postnatal residential history of the study participants, as well as preschool and school addresses, hours spent in preschools and at schools, and the transportation mode used (see Supplementary File S2). It is a pencil-and-paper questionnaire. The collected addresses are being geocoded and will be used to assign air pollution estimates, as well as other geographical exposures.

### 2.6. Psychological Evaluation

Cognitive functioning is examined using the Stanford-Binet Intelligence Scales, 5th edition (SB5, [41] and tests from the Diagnostic Battery for Cognitive Functions Evaluation PU1, [42]). These tests measure attention, working memory, and executive function, which represent domains relevant to both classroom learning and ADHD [43]. Guardians complete the Polish adaptations of the Third Edition of Conners’ Rating Scales [44] and Child Behaviour Checklist (CBCL) for Ages 6–18 [45], providing information on ADHD and other externalising and internalising behavioural issues. Children complete the Polish adaptation of Youth Self-Report (YSR) [46], which is a child-completed version of the CBCL [45,47].

Social functioning is assessed using two questionnaires. The first is the Polish adaptation of the Family Adaptation and Cohesion Evaluation Scales (FACES-IV) questionnaire (Skala Oceny Rodziny; SOR) [48], which measures family function, communication, and family satisfaction. The SOR is completed by both guardians and children. The second is the Polish Siblings Relationship Questionnaire (Kwestionariusz Relacji z Rodzeństwem W Okresie Adolescencji; KRR) [49], which is completed only by children with siblings. 

### 2.7. Behavioural Tasks

The attention network task (ANT) measures the efficiency of the three attentional networks—orienting, alerting, and executive functioning [50]. The ANT demonstrates high immediate test–retest reliability, and scores are not correlated across individuals, suggesting that each network efficiency can be measured somewhat independently [51]. We use the children’s version of the ANT, with timing and task stimuli operationalised identically to those used in [50] with the exception that, since our subjects are older, cue duration is set to 100 ms (as in the adult version). 

The continuous performance test (CPT) is a variant of the Go/NoGo task which allows quantification of deficits in attentiveness, impulsivity, activation/arousal, and vigilance. The CPT has high three-months test–retested reliability [52] and has been validated in children with ADHD and in healthy controls [53]. During the task, geometric figures appear (250 ms) on the computer screen in 18 consecutive blocks of 20 trials (360 trials total). The geometric figures are identical to those used subsequently in the functional MRI (fMRI) variant of the Go/NoGo task. The interstimulus interval (ISI) is set to either 1, 2, or 4 s during each block. Participants are asked to press the spacebar in response to each letter presentation, except for the square and the circle [54]. The proportions of Go to NoGo trials are set at 80% and 20%, respectively [55]. 

All children complete practice runs of both tests until their performance reaches 90% accuracy.

### 2.8. Neuroimaging

All neuroimaging data are acquired at the Małopolska Centre of Biotechnology, Jagiellonian University in Kraków, Poland, on a Siemens MAGNETOM Skyra 3T MRI scanner (Erlangen, Germany) using a 64-channel head coil. Participants are familiarised with the scanner and trained to remain still using a mock scanner (https://pstnet.com/, accessed on 23 December 2021). The mock scanning session lasts for 10 min. During the scan, head movements are constrained by inflatable Pearltec pillows (www.pearl-technology.ch, accessed on 23 December 2021). 

The protocol includes T1-weighted (T1-w), T2-weighted (T2-w), and magnetization-prepared 2 rapid acquisition gradient echo (MP2RAGE) structural images, diffusion-weighted imaging (DWI), two resting-state fMRI (rsfMRI), and two task fMRI runs (see Table 2 for detailed acquisition parameters and order). The scanning protocol, including instructions, lasts for one hour. 

The acquisition sequences for T1-w, T2-w, and fMRI neuroimaging data were adopted from the Adolescent Brain Child Development (ABCD) project [56].

The DWI acquisition sequence was adapted from the UK Biobank (www.ukbiobank.ac.uk, accessed on 23 December 2021) to fit the study scanner and includes multiple b-value volume acquisitions. For the fMRI and DWI acquisitions, we use multi-band accelerated echo planar imaging (EPI) pulse sequences developed by the Center for Magnetic Resonance Research at the University of Minnesota [57,58].

The T1-w sequence provides a better contrast-to-noise ratio in white matter and is used for cortical and subcortical segmentation of the brain [59]. The T2-w sequence allows for the discrimination of structural differences in cerebral fluid-filled regions. The combined use of T1-w/T2-w allows the generation of ratio-based cortical myelin maps [60]. It also provides the anatomical reference for rsMRI and task fMRI data, including anterior–posterior commissure alignment. For improved quality, the T1-w and T2-w protocols include volumetric navigators for prospective motion correction [61]. MP2RAGE achieves a spatially uniform contrast [62] and is used for morphometric analyses of brain anatomy and for construction of cortical myelin maps. DWI enables visualisation and characterisation of white matter tracts [63]. rsfMRI imaging allows for the inference of the intrinsic organization of large-scale brain networks [64]. 

Throughout the structural, MP2RAGE, and DWI acquisitions, participants watch neutral-valence movies (e.g., nature documentaries on the life of birds); throughout the rsfMRI acquisition, participants are instructed to stay awake, to keep still, and to blink normally while looking at a fixation cross.

Finally, task fMRI analyses can target task-dependent whole-brain activity and functional connectivity [65,66]. Based on our hypotheses, we chose an fMRI task that measures response inhibition as a measure of executive functioning, as inhibitory control is positively associated with cognitive flexibility and problem-solving skills and, thus, represents an important functional domain during brain maturation [67]. In addition, diminished inhibitory control is associated with disorders of impulse control, including ADHD [68,69]. This task is identical to the conditioned approach response inhibition task (CARIT) used in the Human Connectome Project–Development (HCP–D) project [70] and is a variant of the Go/NoGo task. The task allows mapping of differential neuronal activity when response inhibition demands are high (NoGo trials) as compared to free prepotent motor execution (Go trials) [52]. The task uses a rapid event-related fMRI design with jittered inter-trial intervals (1000–4500 mm) and randomized inter-target intervals to optimize statistical efficiency [71]. During each run, participants view shape stimuli (*n* = 92) and are instructed to press a button as quickly as possible in response to every shape (Go; *n* = 68) except for a circle and a square (NoGo; *n* = 24) [70,72]. 

Region-of-interest measures will be exported from appropriate MRI analysis packages, including FreeSurfer (http://surfer.nmr.mgh.harvard.edu/; accessed on 23 December 2021), Statistical Parametric Mapping (SPM) 2 (www.fil.ion.ucl.ac.uk/spm, accessed on 23 December 2021), and the Oxford Centre for Functional MRI of the Brain Software Library (FSL; https://fsl.fmrib.ox.ac.uk, accessed on 23 December 2021), and analysed in R [36] using logistic regression, with common best statistical practices such as applying corrections for multiple comparisons. Whole-brain measures will be calculated within the MRI analysis packages themselves and statistical tests appropriate to each imaging modality will be applied. For example, for task fMRI data, we will apply a voxel-wise threshold of either *p* < 0.01 or *p* < 0.001 and an appropriate cluster-level threshold (*p* < 0.05, false discovery rate, FDR).

### 2.9. Air Pollution Exposure Assessment

Three health-relevant air pollutants are considered for this study: PM_2.5_, PM_10_, and NO_2_. Annual and monthly grids of air pollutant concentrations will be created for the study area for the period from 2007 to 2022 using hybrid land use regression (LUR) models, following methods developed by de Hoogh and colleagues [73,74]. Interestingly, the research evaluating the performance of various regression models, regularization techniques, and machine learning methods for air pollution spatial modelling has shown relatively small differences in the prediction of accuracy in air concentration between various algorithms [75].

**Figure 4:**
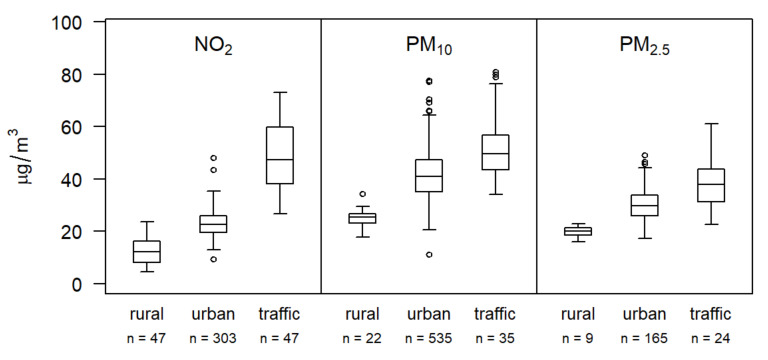
Boxplots illustrating annual means of daily measured NO_2_, PM_10_, and PM_2.5_ air pollutants, grouped by type of station (urban, traffic, rural) from 2007 to 2019, based on monitoring stations in the study area. Between 2007 and 2019, the annual mean values of air pollutant concentrations at the monitoring stations ranged from 4.5 to 73.1 µg/m^3^ for NO_2_, from 11.2 to 80.9 µg/m^3^ for PM_10_, and from 16.1 to 61.1 µg/m^3^ for PM_2.5_.

The LUR models are estimated and validated using measurements from the air quality stations for each of the air pollutants and for each year separately, and will also include monthly LUR models for the prenatal period. Routine measurements of these air pollutants are made by Polish Chief Inspectorate of Environmental Protection via a network of air quality monitoring stations [76]. The locations of the monitoring stations (*n* = 179) in the study area are illustrated in Figure 1. Measured air pollution concentrations between 2007 and 2019 are depicted in Figure 4.

Predictor variables for the LUR models are summarized in Table 3, and detailed in Supplementary Table S1. We calculate atmospheric dispersion of air pollutants according to meteorological conditions using the methodology described in [77,78], providing 1-h concentrations of pollutants. We use satellite images to inform about land use changes. Data processing is completed with QGIS geographic information system (GIS) software [79]. 

Land cover predictor variables are calculated for seven girds at the following resolutions: ~4 km × 4 km, 2 km × 2 km, 1 km × 1 km, 500 m × 500 m, 250 m × 250 m, 125 m × 125 m, and 62.5 m × 62.5 m. 

Exploratory and correlation analyses of average air pollutant concentrations from the monitoring stations and the predictor variables are performed to assess temporal and spatial distribution patterns. Optimal resolution for each type of variable applied in the LUR air quality modelling is determined based on physical interpretability and supported with statistical analyses, including machine learning. Predictor variables are included in the LUR model only if they adhere to the predefined direction of effect.

Models are validated with out-of-sample and cross-validations methods using data from the monitoring stations. Model assumptions are checked through the analysis of residuals, as well as visual inspection of quantile–quantile plots. Log transformations are tested to minimize the effect of non-normal errors, and *p*-values are calculated assuming normal distributions of errors. Kriging of air pollution concentrations will be performed, as it is expected to significantly improve model performance of linear regression algorithms for spatial data [75]. All LUR modelling is conducted in R statistical software [36].

Finally, estimates resulting from the LUR models will be assigned to all residential and preschool/school addresses of each study participant. Prenatal, early postnatal, concurrent, and life-long air pollution estimates will be calculated.

### 2.10. Planned Analyses

The main outcomes resulting from psychological evaluation, behavioural testing, and neuroimaging are listed in Table 4.

We are planning to publish several papers using prenatal, early life, and lifetime PM_2.5_, PM_10_, and NO_2_ as exposures. The papers will be grouped by outcome domain:

1. Structural brain measures
2. Fractional anisotropy
3. Brain measures of inhibitory and attentional functions assessed with task fMRI
4. Resting state connectivity
5. Measures of attention assessed by behavioural tasks
6. ADHD and other externalizing behaviours
7. IQ

Regression analyses will be used to analyse each exposure–outcome pair separately. We will individually assess associations with every pollutant at every exposure window. We will also consider adjusting our models for residuals of co-pollutants, given that PM and NO_2_ tend to be strongly correlated. Confounder sets will be selected using directed acyclic graphs (DAGs) [80] to avoid overadjustment. 

## 3. Conclusions

The primary objective of the NeuroSmog study is to combine state-of-the-art multimodal neuroimaging, psychological assessment, environmental epidemiology, and air pollution modelling to determine the impact of air pollutants on neurophysiological and behavioural outcomes in healthy children and in atypically developing children diagnosed with ADHD. NeuroSmog is based on an ethnically, racially, and culturally homogeneous Polish population, which reduces potential confounding. 

NeuroSmog is the first large-scale study to provide collateral measures of brain structure, function, and connectome, with periodically stratified and lifespan (including prenatal) exposures to air pollutants, and comprehensive child psychological assessments. We use a single MRI scanner and a narrow age range of participants to eliminate multisite acquisition confounding and to reduce epiphenomena related to developmental and hormonal changes in children. Additionally, we have adapted our protocol to fit two other large neuroimaging studies, the ABCD and the HCP-D, and we will make our data openly available to the scientific community providing a rich and easily comparable resource. A particular novelty of NeuroSmog is the inclusion of an fMRI task that directly tests the functioning of neural systems for inhibitory control that are putatively affected by air pollution exposure.

The few neuroimaging air pollution studies conducted to date were done in cities with relatively clean air. Performing this work in southern Poland allows us to investigate the impact of air pollution at concentrations and ranges greater than studied previously, thus increasing the chances of discovering associations, if present. Compared to existing similar studies, the project is unique in terms of its case–control design. Children with ADHD are hypothesized to be more vulnerable to the effects of air pollution. Finally, high-resolution air pollution grids will be produced for the study area using state-of-the-art techniques. We are, thus, confident that the data generated by our project, when combined with appropriate analysis and interpretation, will yield new insights into factors that impact or alter neurodevelopmental trajectories in children.

## 4. Challenges

The COVID-19 pandemic, and disruptions to work, movement, and education that are associated with it, have greatly complicated the fieldwork component of the study. Even under normal conditions, the logistics of managing fieldwork across multiple centres are challenging. In general, all fieldwork has required more effort than originally anticipated and is taking longer than planned. The pandemic has also impacted negatively on response rates: 50% of contacted schools declined to collaborate and outreach towards the remaining 50% remains challenging.

Use of MRI in research presents additional challenges. Some members of the public distrust MRI and, on occasion, it has been difficult to motivate families of both cases and controls to participate in the study. A mixed attitude towards research involving underaged participants, exacerbated by the recent rise of anti-vaccine sentiment, also poses a challenge.

Although we will be able to assess lifelong exposure to air pollution in our study, we are only able to investigate the associations with a limited number of air pollutants. Our PM measures are gross, and we have no knowledge of its actual composition, which could vary considerably across Poland. There are a host of environmental stressors that could potentially affect neurodevelopment and be associated with air pollutants (and therefore cause confounding). The study would be better able to capture some of the wide array of environmental chemicals which might affect neurodevelopment if we had biological samples. Finally, as with all case–control studies, we anticipate that recall bias may affect our analyses, particularly in terms of early life factors and residences.

## Figures and Tables

**Table 1:** NeuroSmog study towns by type.

Air Pollution Level	Population Size	
	Large	Small
**High**	Kraków	Pszczyna
		Czechowice-Dziedzice
		Chrzanów
		Skawina
		Bochnia
**Medium**	Częstochowa Jaworzno	Olkusz
		Żywiec
		Trzebinia
		Nowy Targ
		Kędzierzyn-Koźle
		Strzelce Opolskie
**Low**	Bielsko-Biała	Zakopane
		Cieszyn
		Kłobuck

**Table 2:** Neuroimaging scanning parameters.

Sequence	Matrix	Slices	FOV	% FOV Phase	Resolution (mm)	TR (ms)	TE (ms)	TI (ms)	Flip Angle (deg)	Parallel Imaging	Multi Band Acceleration	Phase Partial Fourier	Diffusion Directions	b-Values	Acquisition Time
**T1-w**	256 × 256	176	256 × 256	100%	1.0 × 1.0 × 1.0	2500	2.88	1060	8	2×	Off	Off	N/A	N/A	06:09
**T2-w**	256 × 256	176	256 × 256	100%	1.0 × 1.0 × 1.0	3200	565	N/A	Variable	2×	Off	Off	N/A	N/A	05:34
**fMRI**	90 × 90	60	216 × 216	100%	2.4 × 2.4 × 2.4	800	30	N/A	52	Off	6	Off	N/A	N/A	2 × 4.11 (task fMRI) 2 × 6.08 (rsfMRI)
**DWI**	104 × 104	72	210 × 210	100%	2.0 × 2.0 × 2.0	3800	101	N/A	78	Off	3	6/8	117	0 (10 dirs) 500 (18-dirs) 1250 (36-dirs) 2500 (53-dirs)	07:31
**MP2RAGE**	256 × 256	176	256 × 256	100%	1.0 × 1.0 × 1.0	5000	3	700	4	3x	Off	Off	N/A	N/A	08:22

DWI—Diffusion-weighted imaging; fMRI—Functional magnetic resonance imaging; FOV—field of view; MP2RAGE—Magnetization-prepared 2 rapid acquisition gradient echo; rsfMRI—Resting-state functional magnetic resonance imaging; T1-w—T1-weighted; T2-w—T2-weighted; TE—echo time; TI—inversion time; TR—repetition time.

**Table 3:** Predictor variables for the air quality LUR modelling.

Type	Source	Main Indicators	Characteristics
Emissions	National Centre for Emissions Management [76], regional data from the voivodeship authorities, emission sector maps	Traffic emissions of air pollutants Residential emission of air pollutants	Daily data available, monthly/annual means incorporated in LUR
Land use	Corine Land Cover	Forest and wooded area Residential area Surface water Vegetation and agricultural area Area under roads, rail, and airport roads Unused land, landfill, excavation Remaining, undeveloped area	Annual data collection years: 2006, 2012, 2018
Road data	Database of Topographic Objects, 1:10, 000 scale, nationwide (BDOT10k)	Type of road (e.g., highway, expressway, main road, etc.) Number of traffic lanes within one road	Data collection from years 2019 to 2020
Air quality	Atmospheric dispersion models [74]	Estimates from the dispersion modelling	Hourly data available, monthly/yearly means incorporated in LUR
Meteorological conditions	Institute of Meteorology and Water Management National Research Institute	Temperature Wind speed and direction Precipitation Relative humidity Atmospheric pressure	Hourly data available, monthly/yearly means incorporated in LUR

BDOT10k—Baza Danych Obiektów Topograficznych (Database of Topographic Objects) at accuracy level 1:10,000; LUR—land use regression model.

**Table 4:** List of the main outcomes of the study.

Tool	Main Outcomes
**Neuroimaging**	
T1-w and T2-w	Volume of subcortical structures
	Cortical grey matter thickness and surface
DWI	Fractional Anisotropy in regions of interest (tractography)
	NODDI
Task fMRI	BOLD activation in the NoGo > Go contrast in the CARIT
	Amplitude of BOLD signal change between task-related and default-mode-network activations
Resting-state connectivity	Functional connectivity in regions of interest
T1-w, T2-w, and MP2RAGE	Cortical myelin content
**Behavioural tasks**	
CPT and CARIT	Omission errors
	Commission errors
	Mean reaction time
	Standard deviation of reaction time
ANT	Mean reaction time
	Alerting network
	Orienting network
	Executive network
**Psychological evaluation**	
CBCL and YSR	Total problems
	Internalizing problems
	Externalizing problems
	Withdrawn/depressed
	Somatic complaints
	Anxious/depressed
	Social problems
	Thought problems
	Attention problems
	Rule-breaking behaviour
	Aggressive behaviour
SB5	General IQ
	Verbal IQ
	Non-verbal IQ
PU1	Selective attention
	Memory–phonological loop
	Visual–spatial memory
	Executive functions
Opinions of assessing psychologists, Conners 3, PU1, SB5, CBCL, and validation	ADHD diagnosis

ADHD—Attention deficit hyperactivity disorder; ANT—attention network test; BOLD—Blood oxygen level dependent effect; CARIT—Conditioned approach response inhibition task; CBCL—Child Behaviour Checklist; CPT—Continuous performance test; DWI—CDiffusion-weighted imaging; fMRI—Functional magnetic resonance imaging; IQ—Intelligence quotient; MP2RAGE—Magnetization-prepared 2 rapid acquisition gradient echo; NODDI—Neurite orientation dispersion and density; PU1—Bateria diagnozy funkcji poznawczych PU1: Pamięć—uwaga—funkcje wykonawcze (Diagnostic Battery for Cognitive Functions Evaluation); SB5—Stanford-Binet Intelligence Scales, 5th edition; T1-w—T1-weighted; T2-w—T2-weighted; YSR—Youth Self-Report.

## Data Availability

The data collected in this study will be available on request from the correspondin author. The data are not publicly available due to the local ethical and legal restrictions.

## Supplementary Materials

The following are available online at https://www.mdpi.com/article/10.3390/ijerph19010310/s1, Supplementary File S1: NeuroSmog–General Questionnaire, Supplementary File S2: NeuroSmog–Address Questionnaire; Supplementary Table S1: Predictor variables for the NeuroSmog LUR models.

## Abbreviations

ABCD	Adolescent Brain Child Development study
ACC	Anterior cingulate cortex
ADHD	Attention deficit hyperactivity disorder
ANT	Attention network test
ASD	Autism spectrum disorder
BDOT10k	Baza Danych Obiektów Topograficznych (Database of Topographic Objects) at accuracy level 1:10,000
BOLD	Blood oxygen level dependent effect
CARIT	Conditioned approach response inhibition task
CBCL	Child Behaviour Checklist
CI	Confidence interval
CPT	Continuous performance test
DAG	Directed acyclic graph
DPLFC	Dorsolateral prefrontal cortex
DWI	Diffusion-weighted imaging
EPI	Echo planar imaging
EU	European Union
FDR	False discovery rate
FOV	Field of view
GIS	Geographic information system
fMRI	Functional magnetic resonance imaging
HCP–D	Human Connectome Project–Development
IQ	Intelligence quotient
ISI	Interstimulus interval
KRR	Kwestionariusz Relacji z Rodzeństwem W Okresie Adolescencji (Syblings Relationship Questionnaire)
LUR	Land use regression model
MP2RAGE	Magnetization-prepared 2 rapid acquisition gradient echo
MRI	Magnetic resonance imaging
NIH	National Institute of Health
NO_2_	Nitrogen dioxide
NODDI	Neurite orientation dispersion and density
PM	Particulate matter
PM_2.5_	Particulate matter with aerodynamic diameter <2.5 µm
PM_10_	Particulate matter with aerodynamic diameter <10 µm
PU1	Bateria diagnozy funkcji poznawczych PU1: Pamięć—uwaga—funkcje wykonawcze (Diagnostic Battery for Cognitive Functions Evaluation)
rsfMRI	Resting-state functional magnetic resonance imaging
SB5	Stanford-Binet Intelligence Scales, 5th edition
SES	Socio-economic status
SMA	Supplementary motor area
SOR	Skala Oceny Rodziny (Family Adaptation and Cohesion Evaluation Scales (FACES IV))
SPM	Statistical parametric mapping
T1-w	T1-weighted
T2-w	T2-weighted
TI	Inversion time
TR	Repetition time
TE	Echo time
WHO	World Health Organization
YSR	Youth Self-Report
