# Supplementary material for: NeuroSmog: Determining the Impact of Air Pollution on the Developing Brain: Project Protocol
Source: Int J Environ Res Public Health. 2021 Dec 28;19(1):310. doi: 10.3390/ijerph19010310 (PMC8744611; doi:10.3390/ijerph19010310)
Supplement: Supplementary file 1 [file ijerph-19-00310-s001.zip › Supplementary File S1.pdf]

Please provide your child’s ID: …………………………………

NeuroSmog – General Questionnaire

NEUROSMOG - PARENTAL QUESTIONNAIRE

We are researching whether air pollution can affect the development of children's brains. To do this, we need to know about other things that might influence brain development and that are related to pollution of outdoor air. Please try to answer the questions in this questionnaire as accurately as possible. This questionnaire consists of 78 questions organized into 8 blocks. It takes approximately 20-25 minutes to fill in. **All questions that mention "your child" refer to the child for whom you gave given consent to participate in the NeuroSmog study.**

Please provide answers by entering "X" in the appropriate box.

BLOCK A – BACKGROUND INFORMATION

1. Date of birth of your child:
   Day: ……….. Month: ……….. Year: ………..

2. Sex of your child:
   - ☐ Female
   - ☐ Male
   - ☐ Other (which?)……………………………….

3. Education of your child's **mother**
   - ☐ Primary education
   - ☐ Secondary education
   - ☐ Higher education
   - ☐ Other (which?) ……………………………….
   - ☐ Don't know / Don't want to answer

4. Education of your child's **father**
   - ☐ Primary education
   - ☐ Secondary education
   - ☐ Higher education
   - ☐ Other (which?) ……………………………….
   - ☐ Don't know / Don't want to answer

5. Please rate how well your family was doing financially **in 2019**
   - ☐ It was very difficult
   - ☐ It was quite difficult
   - ☐ We just managed to make ends meet
   - ☐ We were doing alright
   - ☐ We were living comfortably
   - ☐ Don't want to answer

6. Please rate how well your family was doing financially **during pregnancy with your child and during her or his first years of life**
   - ☐ It was very difficult
   - ☐ It was quite difficult
   - ☐ We just managed to make ends meet
   - ☐ We were doing alright
   - ☐ We were living comfortably
   - ☐ Don't want to answer

7. How many people is living in your child's **current** home, **excluding your child?** *(Please include yourself)*

   ………………………………..

8. Please provide information on all those who is living in your child's **current** home *(including yourself)*

	Age (in years)	Sex *(male, female, other)*	Relation to your child *(parent, step-parent, sibling, grandparent, aunt/uncle, cousin, other)*
Person 1			
Person 2			
Person 3			
Person 4			
Person 5			
Person 6			
Person 7			
Person 8			
Person 9			
Person 10			

BLOCK B – PREGNANCY AND EARLY LIFE OF YOUR CHILD

9. Was the pregnancy with your child a **multiple pregnancy** *(e.g. twins or triplets)*?
   - ☐ Yes
   - ☐ No
   - ☐ Don't know

10. Was your child delivered by **Caesarean section**?
    - ☐ Yes
    - ☐ No
    - ☐ Don't know / Don't want to answer

11. Was your child the **mother's first-born** child?
    - ☐ Yes
    - ☐ No
    - ☐ Don't know

12. Do you know the **gestational age** of your child at birth? *[weeks]*
   - ☐ Yes *(please enter)* ……………………………….
   - ☐ No

13. Do you know the **birthweight** of your child? *[grams]*
   - ☐ Yes *(please enter)* ……………………………….
   - ☐ No

14. Do you know **Apgar score** of your child? *[0 to 10]*
   - ☐ Yes *(please enter)* ……………………………….
   - ☐ No

15. Was your child exclusively breastfed **for the first 4 months** of life?
   (Exclusive breastfeeding means your child received only breast milk and no other liquids or solids with the exception of medicine.)
   - ☐ Yes
   - ☐ No
   - ☐ The child was partially breastfed
   - ☐ Don’t know / Don’t want to answer

16. How often did your child’s **mother** consume alcohol while pregnant with your child?
   - ☐ Never
   - ☐ Almost never
   - ☐ Once per month
   - ☐ Once per week
   - ☐ More than once per week
   - ☐ Every day
   - ☐ Don’t want to answer

17. How often did your child’s **father** consume alcohol while the mother was pregnant with your child?
   - ☐ Never
   - ☐ Almost never
   - ☐ Once per month
   - ☐ Once per week
   - ☐ More than once per week
   - ☐ Every day
   - ☐ Don’t want to answer

18. How often did your child’s **mother** smoke tobacco (or use nicotine patches or nicotine chewing gums) while pregnant with your child?
   - ☐ Never
   - ☐ Almost never
   - ☐ Once per month
   - ☐ Once per week
   - ☐ More than once per week
   - ☐ Every day
   - ☐ Don’t want to answer

19. How often did your child’s **father** smoke tobacco (or use nicotine patches or nicotine chewing gums) while the mother was pregnant with your child?
   - ☐ Never
   - ☐ Almost never
   - ☐ Once per month
   - ☐ Once per week
   - ☐ More than once per week
   - ☐ Every day
   - ☐ Don’t want to answer

20. How often does anyone smoke at your child’s home **currently**? *(including smoking on a balcony or terrace)*
   - ☐ Never
   - ☐ Almost never
   - ☐ Once per month
   - ☐ Once per week
   - ☐ More than once per week
   - ☐ Every day
   - ☐ Don’t want to answer

21. How often did anyone smoke at your child’s home **during the first 5 years** of life? *(including smoking on a balcony or terrace)*
   - ☐ Never
   - ☐ Almost never
   - ☐ Once per month
   - ☐ Once per week
   - ☐ More than once per week
   - ☐ Every day
   - ☐ Don’t want to answer

22. Was your child's mother **physically active** during pregnancy with your child *(e.g. brisk walking, swimming, jogging, yoga, dancing)*?

☐ Yes, throughout the entire pregnancy
☐ Yes, in the early part of pregnancy only
☐ Yes, in the latter part of pregnancy only
☐ No
☐ Don’t know / Don’t want to answer

23. Was your child's mother **doing paid work outside of the home** during pregnancy with your child?

☐ Yes, throughout the entire pregnancy
☐ Yes, in the early part of pregnancy only
☐ Yes, in the latter part of pregnancy only
☐ No
☐ Don’t know / Don’t want to answer

BLOCK C – GENERAL HEALTH OF YOU CHILD

24. Has your child gone through a growth spurt (associated with puberty)?
   - ☐ Not yet
   - ☐ Barely started
   - ☐ Definitely started to happen, but still growing
   - ☐ Seems to be complete
   - ☐ Don’t know / Don’t want to answer

25. Has your child started to grow body hair (e.g., under arms)?
   - ☐ Not yet
   - ☐ Barely started
   - ☐ Definitely started to happen, but is not fully grown
   - ☐ Seems to be fully grown
   - ☐ Don’t know / Don’t want to answer

26. Have you noticed any skin changes in your child, especially spots?
   - ☐ Not yet
   - ☐ Changes have barely started
   - ☐ Changes have definitely started, but are not finished
   - ☐ Changes seem to be complete
   - ☐ Don’t know / Don’t want to answer

Please only answer these questions ***(27 i 28) if your child is male.*** *Otherwise, please skip them and go to the question* ***29.***

27. Has the voice of your child started to change?
    - ☐ Not yet
    - ☐ Voice change has barely started
    - ☐ Voice change is definitely happening, but is not complete
    - ☐ Voice change seems to be complete
    - ☐ Don't know / Don't want to answer

28. Has your child started to grow facial hair (beard or moustache)?
    - ☐ Not yet
    - ☐ Facial hair growth has barely started
    - ☐ Facial hair growth has definitely started (enough to shave)
    - ☐ Facial hair probably grows as fast now as it ever will
    - ☐ Don't know / Don't want to answer

Please only answer these questions ***(29, 30 i 31) if your child is female****. Otherwise, please skip them and go to the question* ***32****.*

29. Have the breasts of your child begun to grow?
    - ☐ Not yet
    - ☐ Breast growth has barely begun
    - ☐ Breast growth has definitely started but is not complete
    - ☐ Breast growth seems to be complete
    - ☐ Don't know / Don't want to answer

30. Has your child begun to menstruate?
    - ☐ No
    - ☐ Yes
    - ☐ Don't know / Don't want to answer

31. *(If the child begun to menstruate)* How old was your child when she first got her period *[years]*

    ……………………………………………

32. How tall is your child? *[cm]*

……………………………………………

33. How much does your child weigh? *[kg]*

……………………………………………

34. Your child is:
    - ☐ Right-handed
    - ☐ Left-handed
    - ☐ Ambidextrous
    - ☐ Don’t know / Don’t want to answer

35. Does your child have any **visual impairment**?
    NOTE: Hyperopia above +6 and myopia below -6 excludes the child from the examination
    - ☐ Yes - corrected
    - ☐ Yes – not corrected
    - ☐ No
    - ☐ Don’t know

36. You indicated that your child has a **visual impairment**. Please specify the dioptre.
    Left eye: …………
    Right eye: ………...

37. Has your child been diagnosed with any **food intolerance**?
    - ☐ No
    - ☐ Yes
    - ☐ Don’t know

If YES please specify food items:

………………………………………………………………………………………………………..

38. Has your child been diagnosed with any **food allergy**?

- ☐ No
- ☐ Yes
- ☐ Don’t know

If YES please specify food items:

………..

39. Has your child been diagnosed with **asthma**?
    - ☐ No
    - ☐ Yes
    - ☐ Don’t know

40. Has your child been diagnosed with **allergic rhinitis** or **hay fever**?
    - ☐ No
    - ☐ Yes
    - ☐ Don’t know

41. Has your child been diagnosed with **eczema** or **atopic dermatitis**?
- ☐ No
- ☐ Yes
- ☐ Don’t know

42. Has your child been diagnosed with **dyslexia**?
- ☐ No
- ☐ Yes
- ☐ Don’t know

43. Has your child been diagnosed with **dysgraphia**?
- ☐ No
- ☐ Yes
- ☐ Don’t know

44. Has your child been diagnosed with **dyscalculia**?
- ☐ No
- ☐ Yes
- ☐ Don’t know

45. Do any of your child's closest relatives (parents, grandparents, siblings, aunt or uncle) have or have had difficulties such as dyslexia/dysgraphia, ADHD, autism, tics?
- ☐ No
- ☐ Yes
- ☐ Don’t know / Don’t want to answer

46. If your child has any **other** diagnosed medical conditions, please write them down here:

………

47. Does your child **currently** have sleeping problems?
- ☐ No
- ☐ Yes
- ☐ Don’t know / Don’t want to answer

48. What kind of sleeping problems? (Please specify ALL options that apply.)
   - ☐ Problems accepting evening bed time
   - ☐ Problems falling asleep
   - ☐ Problems with breathing during sleep
   - ☐ Problems with sleeping through the night
   - ☐ Problems getting up in the morning
   - ☐ Problems with drowsiness during the day
   - ☐ Other
   - ☐ Don’t want to answer

BLOK D – HABITS OF YOUR CHILD

49. Please specify **how many hours per day** your child **usually** spends doing the following activities:

Time spent outdoors **in summer**	……………………
Time spent outdoors **in winter**	……………………
Reading books **on paper**	……………………

50. Please specify **how many hours per day** your child **usually** spends doing the following activities and the electronic devices used *(Please choose 'Does not apply' if your child does not spend any time doing a particular activity)*.

In the field “Most commonly used device”, enter the appropriate letter
A – Computer
B – Smartphone
C – Tablet
D – TV
E – Games console
F – Other

	Hours per day	Most commonly used device	Does not apply
Reading books electronically	…………..	…………..	☐
Spending time on social media (e.g. Facebook, Snapchat, Instagram, TikTok)	…………..	…………..	☐
Watching movies, series or TV programs	…………..	…………..	☐
Watching videos on YouTube	…………..	…………..	☐
Playing computer games	…………..	…………..	☐

51. **How often** does your child **usually** do **strenuous** physical activity outside of school that makes her or him get out of breath or sweat more than usual (*e.g., playing team sports, dancing, swimming, etc.*)?
   - ☐ Every day
   - ☐ 4-6 times per week
   - ☐ 1-3 times per week
   - ☐ At least once per month
   - ☐ Less than once per month
   - ☐ Never
   - ☐ Don't know

52. Approximately **how many hours per week** does your child **usually** do **strenuous** physical activity outside of school that makes her or him get out of breath or sweat more than usual *(e.g., playing team sports, dancing, swimming)*?
   - ☐ More than 6 hours per week
   - ☐ 5-6 hours per week
   - ☐ 3-4 hours per week
   - ☐ 1-2 hours per week
   - ☐ Less than 1 hour per week
   - ☐ None
   - ☐ Don't know

53. How many hours does your child **usually** sleep in a typical 24 hour period, including sleep during the night and any naps taken during the day?

   …………………..

54. How often does your child **usually** eat or drink the following:

	Never	Rarely	Several times per month	Several times per week	Daily	Don't know
Meat or poultry, *including sausage*	☐	☐	☐	☐	☐	☐
Fish or seafood	☐	☐	☐	☐	☐	☐
Cod oil	☐	☐	☐	☐	☐	☐
Potatoes *(excluding fries)*	☐	☐	☐	☐	☐	☐
Other vegetables, *excluding potatoes*	☐	☐	☐	☐	☐	☐
Fresh fruits	☐	☐	☐	☐	☐	☐
Porridge	☐	☐	☐	☐	☐	☐
Bread	☐	☐	☐	☐	☐	☐
Fast-food	☐	☐	☐	☐	☐	☐
Food heated in plastic container in microwave	☐	☐	☐	☐	☐	☐
Sugary drinks *(e.g., carbonated drinks, sweet juices)*	☐	☐	☐	☐	☐	☐
Milk	☐	☐	☐	☐	☐	☐
Dairy products *(e.g., cheese, yoghurt)*	☐	☐	☐	☐	☐	☐
Nuts	☐	☐	☐	☐	☐	☐
Sweets *(e.g., candy, ice cream)*	☐	☐	☐	☐	☐	☐

BLOCK E – ACADEMIC PERFORMANCE OF YOUR CHILD

55. What is **the most recent end-of-year** grade your child attained in **mathematics**?

- ☐ 1
- ☐ 2
- ☐ 3
- ☐ 4
- ☐ 5
- ☐ 6
- ☐ Don’t know

56. What is **the most recent end-of-year** grade your child attained in **Polish**?

- ☐ 1
- ☐ 2
- ☐ 3
- ☐ 4
- ☐ 5
- ☐ 6
- ☐ Don’t know

57. What is **the most recent end-of-year** grade your child attained in their **first foreign language**?

- ☐ 1
- ☐ 2
- ☐ 3
- ☐ 4
- ☐ 5
- ☐ 6
- ☐ Don’t know

58. In which school year is your child currently? *If it is vacation at the time of completing this questionnaire - which year did your child complete?*
    - ☐ 4
    - ☐ 5
    - ☐ 6
    - ☐ 7

BLOCK F – HOME OF YOUR CHILD

59. Are any of the following accessibly in your child's **current** home?
60.

	Yes	No	Don't know
Balcony	☐	☐	☐
Terrace	☐	☐	☐
Garden	☐	☐	☐

61. Please mark (circle) the value on the scale that best describes your relationship with the natural environment. **How interconnected are you with nature?** *(„Self" = you, „Nature" = the environment.)*

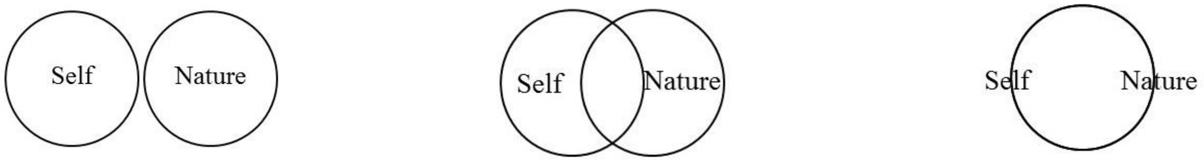

1 ------------- 2 ------------- 3 ------------- 4 ------------- 5 ------------ 6 ------------- 7

62. Are there plants in your child's **current** home, that is, indoor houseplants or plants grown on a balcony or a terrace?
- ☐ Yes
- ☐ No
- ☐ Don't know

63. How many houseplants are there in your child's **current** home??
- ☐ None
- ☐ 1 to 5
- ☐ 6 to 10
- ☐ 11 to 20
- ☐ 21 to 50
- ☐ More than 50

64. Which of the following pets are **currently** kept in your child's home?

	Yes	No	Don't know
Dog	☐	☐	☐
Cat	☐	☐	☐
Rabbit	☐	☐	☐
Hamster	☐	☐	☐
Guinea pig	☐	☐	☐
Bird	☐	☐	☐
Fish	☐	☐	☐
Other	☐	☐	☐

65. Which of the following pets were kept in your child's home at some point **during your child's first 5 years** of life?

	Yes	No	Don’t know
Dog	☐	☐	☐
Cat	☐	☐	☐
Rabbit	☐	☐	☐
Hamster	☐	☐	☐
Guinea pig	☐	☐	☐
Bird	☐	☐	☐
Fish	☐	☐	☐
Other	☐	☐	☐

66. To what extent does **road traffic noise** coming from outside bother, disturb or annoy you, when you are at your child's current home?

Not at all **Extremely**

1 ------ 2 ------ 3 ------ 4 ------ 5 ----- 6 ------ 7 ------ 8 ------ 9 ------ 10

67. How polluted do you think the **air** in your child's neighbourhood is?

Not at all **Extremely**

1 ------ 2 ------ 3 ------ 4 ------ 5 ----- 6 ------ 7 ------ 8 ------ 9 ------ 10

68. Please specify if an **air purifier** is/was in use in your child's home during the following time periods (Please specify ALL options that apply.):
   - ☐ Yes, during pregnancy
   - ☐ Yes, during first 5 years of life
   - ☐ Yes, used currently
   - ☐ No / Other
   - ☐ Don’t know / Don’t want to answer

69. Please specify if an **air humidifier** is/was in use in your child's home during the following time periods (Please specify ALL options that apply.):
   - ☐ Yes, during pregnancy
   - ☐ Yes, during first 5 years of life
   - ☐ Yes, used currently
   - ☐ No / Other
   - ☐ Don’t know / Don’t want to answer

70. For each purpose, please select the **main** heating method **currently** used in your child's home:

	Heating network	Solid fuel (e.g., coal)	Gas	Electricity	Wood	Don’t know
House heating	☐	☐	☐	☐	☐	☐
Water heating	☐	☐	☐	☐	☐	☐
Cooking	☐	☐	☐	☐	☐	☐

71. For each purpose, please select the **main** heating method that was used in your child's home **during the child's first 5 years** of life::

	Heating network	Solid fuel (e.g., coal)	Gas	Electricity	Wood	Don’t know
House heating	☐	☐	☐	☐	☐	☐
Water heating	☐	☐	☐	☐	☐	☐
Cooking	☐	☐	☐	☐	☐	☐

BLOCK G – NEIGHBOURHOD OF YOUR CHILD

72. We are interested to know how safe you consider your child's current neighbourhood to be.
(Please select the answers that best apply to your child's neighbourhood.)

	Strongly disagree	Somewhat disagree	Somewhat agree	Strongly agree	Don't know
The streets are well lit at night	☐	☐	☐	☐	☐
Pedestrians and cyclists can be easily seen by people in their homes	☐	☐	☐	☐	☐
I see and speak to other people when I am out walking	☐	☐	☐	☐	☐
There is a high crime rate	☐	☐	☐	☐	☐
The crime rate makes it unsafe to go on walks **during the day**	☐	☐	☐	☐	☐
The crime rate makes it unsafe to go on walks **at night**	☐	☐	☐	☐	☐

73. We are interested how good or bad are **relations with other people** in your child's current neighbourhood.
(Each row below refers to two opposite situations, one on the left, the other on the right. Please move the slider in each row describing the situation which is closest to your perception. If you cannot choose between the two opposite situations move the slider to "neutral".)

Very strongly **disagree**				Neutr al			Very strongly **agree**	
	-3	-2	-1	0	1	2	3	
Most people in this area **can't be trusted**	☐	☐	☐	☐	☐	☐	☐	Most people in this area **can be trusted**
People in this area **will take advantage of you**	☐	☐	☐	☐	☐	☐	☐	People in this area **will always treat you fairly**
If you were in **trouble**, there is **nobody** in this area who **would help**	☐	☐	☐	☐	☐	☐	☐	If you were in **trouble**, there are **lots of people** in this area who **would help you**
Most people i this area are **unfriendly**	☐	☐	☐	☐	☐	☐	☐	Most people in this area are **friendly**
People in this area have **NO community spirit**	☐	☐	☐	☐	☐	☐	☐	People in this area have **LOTS of community spirit**
People in this area **only look out for themselves**	☐	☐	☐	☐	☐	☐	☐	People in this area d**o things to help the community**
It is **hard to earn people's respect** in this area	☐	☐	☐	☐	☐	☐	☐	People in this area **treat each other with respect**
People in this area **disapprove of others who ae not like them**	☐	☐	☐	☐	☐	☐	☐	People in this area **are tolerant to others who are not like them**
In this area there are **some people who belong and some who don't**	☐	☐	☐	☐	☐	☐	☐	Everybody who lives in this area **belongs just as much as everybody else**

74. We are interested in **green space** in your child's current neighbourhood.
(Please select the answers that best apply to your child's neighbourhood. Please choose ”Does not apply” if a statement is not relevant to your situation.)

	Strongly agree	Agree	Somewhat agree	Neither agree nor disagree	Somewhat disagree	Disagree	Strongly disagree	Does not apply
My child’s neighbourhood is ”green” *(there are street trees, parks, gardens, lawns, etc.)*	☐	☐	☐	☐	☐	☐	☐	☐
Outdoor vegetation can be seen from my child’s windows	☐	☐	☐	☐	☐	☐	☐	☐
The green spaces in my child’s neighbourhood are of good quality	☐	☐	☐	☐	☐	☐	☐	☐
My child spends leisure time in green spaces *(e.g., parks, forests, meadows, gardens)* **in the neighbourhood**	☐	☐	☐	☐	☐	☐	☐	☐
My child spends leisure time in green spaces *(e.g., parks, forests, meadows, gardens)* **outside of the neighbourhood**	☐	☐	☐	☐	☐	☐	☐	☐

75. We are interested in **blue space (water features)** in your child's current neighbourhood.
(Please select the answers that best apply to your child's neighbourhood. Please choose “Does not apply” if a statement is not relevant to your situation.)

	Strongly agree	Agree	Somewhat agree	Neither agree nor disagree	Somewhat disagree	Disagree	Strongly disagree	Does not apply
There are blue spaces *(e.g., a pond, river, canal, fountain)* in my child's neighbourhood	☐	☐	☐	☐	☐	☐	☐	☐
Blue spaces can be seen from the windows of my child's home	☐	☐	☐	☐	☐	☐	☐	☐
The blue spaces in my child's neighbourhood are of good quality	☐	☐	☐	☐	☐	☐	☐	☐
My child spends leisure time near blue spaces **in the neighbourhood**	☐	☐	☐	☐	☐	☐	☐	☐
My child spends leisure time near blue spaces **outside of the neighbourhood**	☐	☐	☐	☐	☐	☐	☐	☐

76. We are interested how **diverse** you consider your child's current neighbourhood to be. Please specify how many different types of **birds** you remember having noticed in your child's neighbourhood

- ☐ None
- ☐ 1-2 different types
- ☐ 3-5 different types
- ☐ 6-10 different types
- ☐ More than 10 different types
- ☐ Don't know

BLOCK H – CONCLUSION

77. This questionnaire has been filled in by...

- ☐ The child’s mother
- ☐ The child’s father
- ☐ Someone else (please specify) ………………………………

78. Please use the box below to provide feedback or any additional information you consider important but which was not asked about in this survey

……………………………………………………………………………………………………..

……………………………………………………………………………………………………..

……………………………………………………………………………………………………..

We thank you for your time spent taking this survey!
