# Supplementary material for: NeuroSmog: Determining the Impact of Air Pollution on the Developing Brain: Project Protocol
Source: Int J Environ Res Public Health. 2021 Dec 28;19(1):310. doi: 10.3390/ijerph19010310 (PMC8744611; doi:10.3390/ijerph19010310)
Supplement: Supplementary file 1 [file ijerph-19-00310-s001.zip › Supplementary File S2.pdf]

NeuroSmog

UNIWERSYTET JAGIELLOŃSKI W KRAKOWIE

IOŚ-PIB
INSTYTUT OCHRONY ŚRODOWISKA
PAŃSTWOWY INSTYTUT BADAWCZY

Participant ID ☐ ☐ ☐ ☐ ☐ ☐ ☐ ☐

ADDRESS QUESTIONNAIRE

In order for us to be able to more accurately assess the possible effects of polluted air on brain development, please provide us with:
1. The addresses where your child has lived since birth;
2. The addresses where your child's mother lived during the pregnancy;
3. The addresses of all schools and preschools your child has attended.

All address data you provide to us will be converted into GPS coordinates and will not be used for any purposes other than our research. The GPS coordinates data will be stored in such way that no one, not even those conducting the research, will be able to identify you or your child.
If you do not remember full addresses, please provide us with all the details known to you.

1. **Your child's home addresses since birth** ***[If your child lived at two addresses at any point in time, please provide both].***

Street and house number	Town	Postcode	Moved in	Lived there until
			_ _/_ _ _ _ MM / YYYY	current
			_ _/_ _ _ _ MM / YYYY	_ _/_ _ _ _ MM / YYYY

			__/____ MM / YYYY	__/____ MM / YYYY
			__/____ MM / YYYY	__/____ MM / YYYY
			__/____ MM / YYYY	__/____ MM / YYYY
			__/____ MM / YYYY	__/____ MM / YYYY
			__/____ MM / YYYY	__/____ MM / YYYY
			__/____ MM / YYYY	__/____ MM / YYYY
			__/____ MM / YYYY	__/____ MM / YYYY
			__/____ MM / YYYY	__/____ MM / YYYY

2. Home addresses of residence of your child's mother during the pregnancy.

Street and house number	Town	Postcode	Moved in	Lived there until
			_ _/_ _ _ _ MM / YYYY	_ _/_ _ _ _ MM / YYYY
			_ _/_ _ _ _ MM / YYYY	_ _/_ _ _ _ MM / YYYY
			_ _/_ _ _ _ MM / YYYY	_ _/_ _ _ _ MM / YYYY
			_ _/_ _ _ _ MM / YYYY	_ _/_ _ _ _ MM / YYYY
			_ _/_ _ _ _ MM / YYYY	_ _/_ _ _ _ MM / YYYY

3. **Addresses of schools and preschools** ***[If you do not remember the addresses, please provide the name/postal code and town where the school is located].***

**Name**	**Street and house number**	**Town**	**Postcode**	**Started attending**	**Stopped attending**	**Average number of hours per week**	**Main mode of transportation** ***(Please circle)***
				__/____ MM / YYYY	Current		Public transport<br>Car/taxi<br>Bicycle<br>On foot<br>Other
				__/____ MM / YYYY	__/____ MM / YYYY		Public transport<br>Car/taxi<br>Bicycle<br>On foot<br>Other
				__/____ MM / YYYY	__/____ MM / YYYY		Public transport<br>Car/taxi<br>Bicycle<br>On foot<br>Other
				__/____ MM / YYYY	__/____ MM / YYYY		Public transport<br>Car/taxi<br>Bicycle<br>On foot<br>Other
				__/____ MM / YYYY	__/____ MM / YYYY		Public transport<br>Car/taxi<br>Bicycle<br>On foot<br>Other

Thank you very much for providing us this indispensable information!
